# Reviving the Art of Onlay Cast Metal Restoration

**DOI:** 10.7759/cureus.63188

**Published:** 2024-06-26

**Authors:** Namrata P Jidewar, Manoj Chandak, Aditya Patel, Shweta Sedani, Mithilesh M Dhamande, Paridhi Agrawal

**Affiliations:** 1 Department of Conservative Dentistry and Endodontics, Sharad Pawar Dental College and Hospital, Datta Meghe Institute of Higher Education and Research, Wardha, IND; 2 Department of Prosthodontics and Crown and Bridge, Sharad Pawar Dental College and Hospital, Datta Meghe Institute of Higher Education and Research, Wardha, IND

**Keywords:** bevels, prosthesis, indirect restoration, onlay, cast metal

## Abstract

Cast metal restorations have been a cornerstone in restorative dentistry for decades, providing durable and reliable solutions for restoring damaged teeth. This case report explores the evolution of cast metal restoration techniques, highlighting recent advancements and their implications in modern dental practice involving indirect cast metal onlay restoration as a successful treatment option that involves the replacement of the tooth cusp and reinforcement of the tooth through indirect restoration. Historically, cast metal restorations, commonly fabricated from alloys such as gold, have offered superior mechanical properties, biocompatibility, and longevity compared to other materials. However, concerns regarding esthetics and cost have prompted the development of alternative materials such as ceramics and composite resins. Nonetheless, cast metal restorations remain indispensable for certain clinical scenarios, particularly in cases of extensive damage or high occlusal forces.

## Introduction

Fracture, caries, and/or mechanical preparation can result in structural tooth loss, a frequent clinical occurrence. The teeth that experience the greatest amount of masticatory load are the mandibular and maxillary molars, which puts them at a higher risk of breaking [1]. Because of their constant high load bearing, functional cusps have been the most often affected cusps, which tend to fracture. Reconstruction of such cases through direct composite or amalgam restorations is not recommended [2]. As a result, indirect restorative techniques employing cast metal restorations such as onlay are required to replace the functional and nonfunctional cusps [3]. Dr. Philbrook introduced inlay dentistry for the first time in 1897. This came when Taggart introduced the cast gold restoration technique [4]. The type of restoration chosen depends on the amount and condition of the remaining tooth structure. Direct restoration is not an option for teeth with significant tooth structure loss because it cannot provide the necessary resistance and retention form. For teeth like this, indirect restorations such as overlays, onlays, and inlays are the answer. The overall function and shape of the damaged tooth are sufficiently supported by these restorations in terms of resistance and retention [5].

## Case presentation

**Figure 1:**
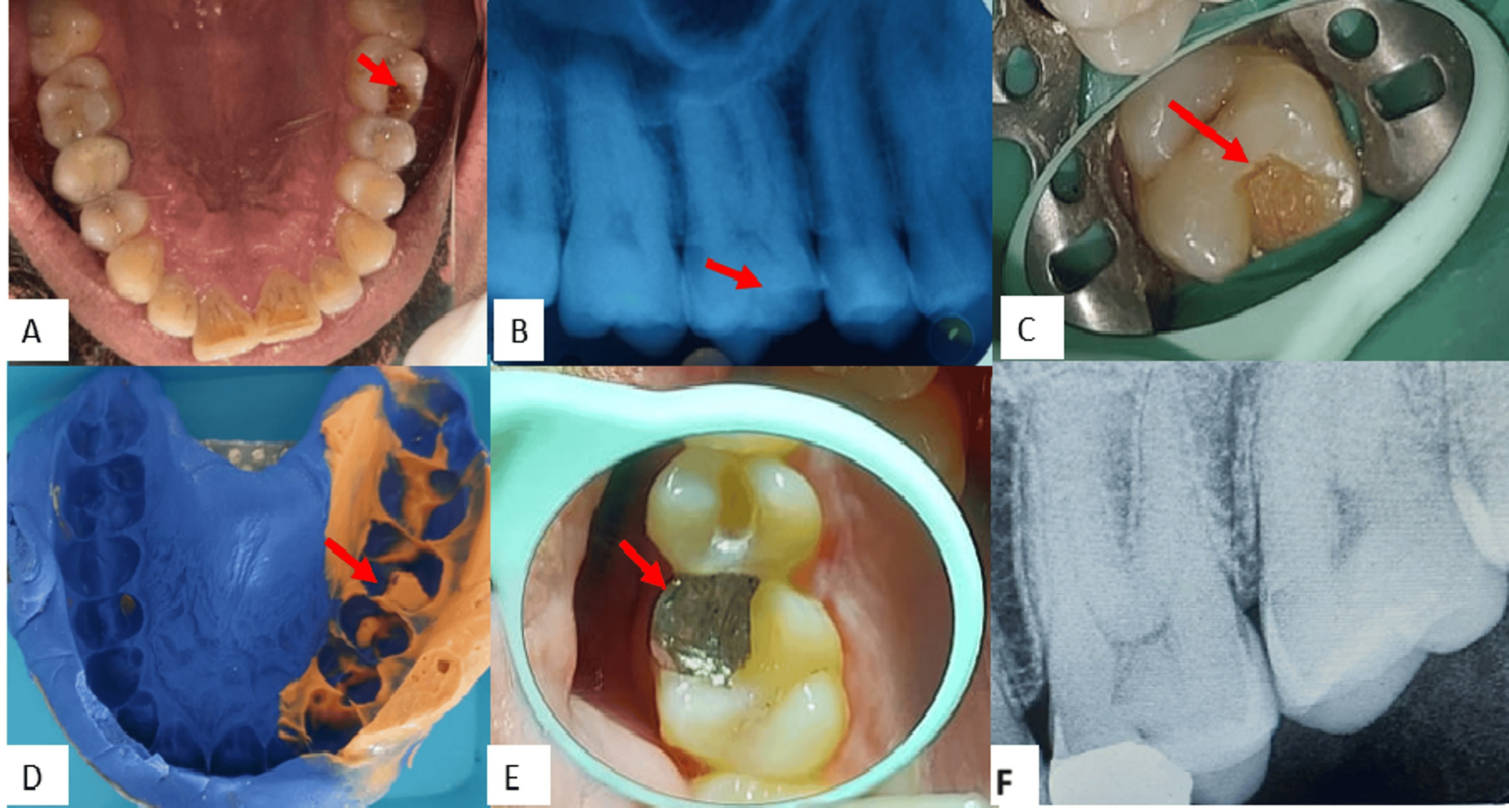
The image shows (A) preoperative intraoral, (B) preoperative radiograph, (C) cavity preparation, (D) elastomeric impression of the upper arch, (E) postoperative picture after cementation, and (F) postoperative radiograph

A 30-year-old man presented to the Department of Conservative Dentistry and Endodontics complaining, primarily, of a restoration that had dislodged in the upper left back region of the jaw for a month. His family background and medical history did not play a role. The same problematic tooth had several restorations in the past, according to the dental history. An intraoral examination revealed 26 cavities and a cracked cusp (Figure 1A). The intraoral periapical radiograph showed radiolucency involving enamel and dentin and not approaching pulp with tooth 26 (Figure 1B). The neural sensibility test showed an early response with 26 EPT (electric pulp test). The diagnosis was symptomatic reversible pulpitis. Figure 1 shows the preoperative, intraoperative, and final cementation of the prosthesis.

In this instance, indirect onlay was the selected course of action. The purpose of the local anesthetic was to minimize sensitivity and discomfort. Rubber dam isolation was carried out, and the occlusal punch cut was accomplished using no. 271 with an initial depth of 1.5 mm, verified with a periodontal probe. Subsequently, the cavity was extended to remove the caries, ensuring that it did not exceed two-thirds of the intercuspal distance and that there were no undercuts for the secondary retention grooves. The Prima Dental Taper Fissure Bur (Plain Cut Long Head, Gloucester, United Kingdom) FG-169L carbide bur was used in the axial wall on the buccal and lingual sides. The occlusal bevel, including the circumferential tie, was prepared with an 8862 bur with fine grit. For the upper arch, elastomeric impression material was used in the wash impression technique (Orikam Neopure A-Silicone Kit Elastomeric Impression Material, Gurgaon, India), and for the lower arch (refer to Figure 1D), alginate impression was used before pouring the cast. After die cutting and casting, a wax pattern was created and invested using type II inlay wax. After that, the metal inlay (made of a base metal alloy of cobalt-chromium) was polished and completed, and the fit was assessed using the cast. This final inlay was then placed in the patient’s mouth and checked for occlusion and high points, followed by final cementation of the restoration using type I luting GIC (GC Gold Label Type 1 Luting and Lining Cement, Tokyo, Japan) (Figure 1E, 1F). With the aid of dental floss and a sharp explorer, the extra cement was eliminated. After cementation, the patient received instructions on oral hygiene and witnessed a demonstration by a model showing proper brushing and flossing techniques.

## Discussion

The oldest restorative material, silver amalgam, has a weak marginal strength and can shatter easily despite its strong compressive strength, which has been demonstrated through testing over time. It is also highly cytotoxic and is prohibited from being used. Its cytotoxicity is dependent on the ability of mercury to modify protein tertiary and quaternary structures, due to which there are varied dose-dependent effects ranging from mild to severe, such as central nervous system (CNS) and cardiovascular system (CVS) depression. Despite the esthetic appeal and ease of use of composite restorations, they consistently undergo polymerization shrinkage, leading to potential hypersensitivity [6-11]. As a result, these choices were excluded.

For large and extensive cavities, such as the one in this case, porcelain fused to metal or a full ceramic crown can be considered. The main drawback is that the ceramic crown requires significant tooth-cutting [7]. For cases such as this, cast metal onlay is regarded as one of the most effective repair options. The strong marginal integrity and compressive strength of the material, which enable it to withstand high masticatory stresses, provide an explanation for this. This time, the same was required for the restoration of 26 shattered teeth. A few other benefits of cast metal onlay are that it is economical, has longevity, is an improved visual aid for margins, and improves the creation and management of contours and contact [8].

According to a recent study conducted by Dhareula et al., both metal and composite indirect restoration showed comparable clinical success [12]. For the restoration to operate properly and last a long time, it is essential to maintain gingival and periodontal health. Over- or under-contouring should be avoided, as it is detrimental to the health of the gingival tissue. Choosing the indirect approach preserves the health of the gingiva and periodontal tissues, as it provides greater control over touch and contouring. Margins were preserved supragingival for the same reasons: they are the most adaptable and promote gingival health [9-12].

## Conclusions

In dentistry, cast metal onlays offer an appropriate treatment that is sometimes disregarded and underutilized. The method yields a very strong and long-lasting restoration, but it does require several patient visits and strong laboratory support. In this instance, cast metal onlay was the recommended course of action because amalgam or composite restorations could not adequately address the clinical circumstances at hand. In addition to maintaining them in operation for years, cast metal restorations offer a means of reconstructing the correct occlusal architecture.
